# Association Between Dental Caries and Obesity among Children with Special Health Care Needs

**DOI:** 10.3290/j.ohpd.b927717

**Published:** 2021-01-28

**Authors:** Roshan Noor Mohamed, Sakeenabi Basha, Yousef Al-Thomali, Fatma Salem AlZahrani, Amal Adnan Ashour, Nada Eid Almutair

**Affiliations:** a Assistant Professor, Department of Pedodontics, Faculty of Dentistry, Taif University, Taif, Saudi Arabia. Concept and design; acquisition, analysis, and interpretation of data; drafted and critically revised manuscript, gave final approval of manuscript, agreed to be accountable for all aspects of work ensuring integrity and accuracy.; b Assistant Professor, Department of Community Dentistry, Faculty of Dentistry, Taif University, Taif, Saudi Arabia. Concept and design; data analysis and interpretation; drafted and critically revised manuscript; agreed to be accountable for all aspects of work ensuring integrity and accuracy.; c Associate Professor, Department of Orthodontics, Faculty of Dentistry, Taif University, Taif, Saudi Arabia. Acquisition, analysis, and interpretation of data; drafted and critically revised manuscript; agreed to be accountable for all aspects of work ensuring integrity and accuracy.; d Assistant Professor, Department of Pedodontics, Faculty of Dentistry, Taif University, Taif, Saudi Arabia. Acquisition, analysis, and interpretation of data; drafted and critically revised manuscript; agreed to be accountable for all aspects of work ensuring integrity and accuracy.; e Associate Professor, Department of Oral Pathology, Faculty of Dentistry, Taif University, Taif, Saudi Arabia. Concept and design; acquisition, analysis, and interpretation of data; drafted and critically revised manuscript; agreed to be accountable for all aspects of work ensuring integrity and accuracy.; f Community Services Coordinator, Department of Community Dentistry, Faculty of Dentistry, Taif University, Taif, Saudi Arabia. Concept and design; acquisition, analysis, and interpretation of data; drafted and critically revised manuscript; agreed to be accountable for all aspects of work ensuring integrity and accuracy.

**Keywords:** dental caries, obesity, special needs

## Abstract

**Purpose:**

Obesity and dental caries constitute an important public health problem worldwide. Special-needs children are at higher risk of developing dental caries and obesity because of their physical, neurological, or behavioural impairment or due to side effects of the medications they take. The present study was conducted to assess the association between dental caries and obesity among children with special health care needs in Taif City, Saudi Arabia.

**Materials and Methods:**

A descriptive cross-sectional study was conducted among 400 (220 girls and 180 boys) special-needs children. Body mass index (BMI) was determined by using height and weight measurements. Dental caries was recorded according to World Health Organization criteria. The association between caries and obesity was assessed using multivariable logistic regression analysis.

**Results:**

289 (72.3%) children presented with caries with mean dmft and DMFT of 3.9 ± 2.7 and 4.8 ± 2.3, respectively. Regression analysis showed specials needs children were at a greater risk of having dental caries: 1.69 times (CI: 0.18–2.62, p < 0.05) greater with obesity; 2.01 (CI: 0.18–3.09, p < 0.05) times greater with sugar consumption; 2.21 times (CI: 1.27–4.12, p < 0.001) greater with cerebral palsy; and 2.27 (CI: 1.29–5.12, p < 0.001) times greater with intellectual disability.

**Conclusion:**

The present study showed a positive association between dental caries and obesity among children of special health care needs. Hence, a focused approach towards the common risk factors is essential to prevent both obesity and dental caries in special-needs children.

Obesity and dental caries constitute an important public health problem worldwide. The global prevalence of childhood obesity varies from 8% to 30%, depending upon the population studied.^9,26^ The prevalence of childhood obesity in Saudi Arabia varies from 13% to 23% depending upon the region studied.^1,4^

The prevalence of caries varies from 70% in permanent dentition with a mean DMFT of 3.5, to 80% in primary dentition with a mean dmft of 5.0.^3,13^ Both caries and obesity show multifactorial etiology, with diet as a common risk factor, and a few researchers have found a positive association between caries and obesity.^5,7^ However, two systematic reviews found contradictory results with no positive association between the two.^22,23^

Special-needs children often require health care interventions based on physical, mental, or behavioral conditions that limit normal activities of daily.^6^ Studies have shown these children are at higher risk of developing caries and obesity because of their physical, neurological, behavioral impairment or due to side effects of the medications they take.^8,14,16-18,29,24^

Although a positive association of cariesw with obesity has been reported,^5,7,19^ no previous studies examined association between obesity and caries among special-needs children, despite their higher risk of developing caries^8,16,21,25^ and obesity.^14,17,18^ The purpose of the present the study was to determine the association between caries, obesity, and type of disability in children with special health-care needs in Taif City, Saudi Arabia.

## Materials and Methods

### Study Design, Sample Selection, and Study Population

The study design was descriptive and cross-sectional, conducted among 400 (220 girls and 180 boys) children with special health care-needs in Taif City, Makkah Province, KSA. The study was conducted from September 2018 to March 2019. A pilot study was conducted among 50 special-needs children to calculate the sample size and check the reliability of questionnaire. Based on the pilot study results, it was predicted that at least 40% of special-needs children would have caries. With this anticipated population proportion of 0.40, at a type I error of 0.5% and a power of 80%, a sample of 400 needed to be recruited for this study. Twenty-five schools were selected randomly from all the five zones of the city (five schools from each zone). The schools were open for all the children and only those with special needs were selected. A two-stage random sampling method was followed, with school as the primary sampling unit and individual special-needs children as unit of inquiry. The study was approved by the institutional review board of Taif University (ethical clearance number – 39-11007-0029). Written informed consent was obtained from the parents/guardians of the participants. Subjects denying written informed consent, refusing cooperation, or exhibiting aggressive behavioural tendencies were excluded from the study.

### Questionnaire

**Fig 1:**
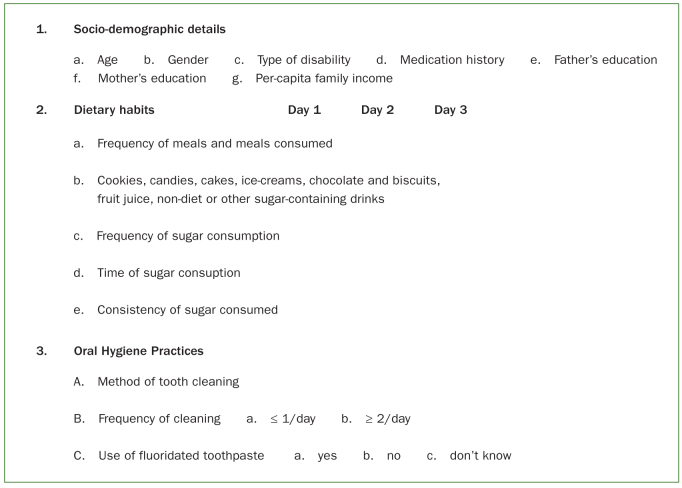
Questionnaire.

A pretested questionnaire was utilised (Cronbach α = 0.80) to collect information on sociodemographic details (age, gender, disability condition), dietary habits, oral hygiene practices, medication history. Dietary information was collected from 72-h recall interviews which spanned different days of the week, including a weekend day and 2 weekdays. The form, frequency, consistency and time of sugar intake were recorded (Fig 1).

### Categorisation of Disability

The disability records were taken from school and categorised into 6 groups: cerebral palsy (CP), autistic disorder (A), Down’s syndrome (DS), intellectual disability (ID), deafness or blindness or both (DB), multiple disabilities or with syndromes (MD).

### Anthropometric Measurements

Body mass index (BMI; weight/height in kg/m^2^) was determined. Children were categorised according to Al-Herbish et al^2^ criteria based on their age and gender into four specified groups: underweight: <5th percentile; normal weight = 5th percentile to <84th percentile; overweight: 85th to <95th percentile; obese: ≥ 95th percentile.

### Oral Examination

All the special-needs children included in the study were examined by a single examiner under natural light using sterile, plane mouth mirrors and CPI probes. The diagnosis of caries (dmft or DMFT/dmfs or DMFS) was made according to the World Health Organization criteria.^28^ The visual and tactile method was used for occlusal lesions and frank cavitation in interproximal lesions to record caries without the use of radiographs. The examiner was calibrated to achieve a statistically significant intra-examiner correlation concerning the diagnostic criteria of caries (Kappa value of 0.85, p < 0.05). Intra-examiner reproducibility was assessed for caries criteria by examining 15% of subjects twice on successive days.

### Statistical Analysis

The difference in proportion was tested using chi-squared tests and Kruskal-Wallis H- followed by the Mann-Whitney U-test for intergroup comparison. The difference in means was tested using Student’s t-test and one-way ANOVA followed by Tukey’s post-hoc analysis for intergroup comparison. Multivariable logistic regression was used to determine the relationships between caries prevalence (yes/no) and obesity. Other factors included gender, age, diet, oral hygiene practice, and type of disability. The analyses were performed using SPSS v 22 (IBM SPSS Statistics, IBM; Armonk, NY, USA). All statistical tests were two-sided, and the significance level was set at p < 0.05.

## Results

Among 400 children, 123 (30.8%) presented with intellectual disability (ID), 107 (26.8%) with autism, 70 (17.5%) with Down’s syndrome, and 43 (10.7%) with cerebral palsy (CP). The mean BMI for the entire study population was 22.3 ± 3.1. One hundred nine (27.2%) children presented with obesity and 77 (19.3%) with overweight (Table 1).

**Table 1:** Body Mass Index (BMI) categories according to variables studied

Variable	BMI categories			
	Underweight n (%)	Normal weight n (%)	Overweight n (%)	Obese n (%)
**Age in years**				
6–11 (n = 160)	9 (5.6)	76 (47.5)	40 (25)	35 (21.9)
12–16 (n = 240)	14 (5.8)	115 (47.9)	37 (15.5)	74 (30.8)
Chi-squared test, p-value	0.12	0.13	0.06	0.06
**Gender**				
Boys (n = 180)	13 (7.2)	86 ( 47.8)	33 (18.3)	48 (26.7)
Girls (n = 220)	10 (4.6)	105 (47.7)	44 (20)	61 (27.7)
Chi-squared test, p-value	0.09	0.13	0.09	0.12
**Type of disability**				
CP (n = 43)	3 (7.0)	22 (51.2)	3 (7.0)	15 (34.8)
A (n = 107)	5 (4.7)	49 (45.8)	15 (14.0)	38 (35.5)
DS (n = 70)	2 (2.9)	42 (60)	15 (21.4)	11 (15.7)
ID (n = 123)	7 (5.7)	59 (47.9)	32 (26.1)	25 (20.3)
DB (n = 33)	3 (9.1)	10 (30.3)	8 (24.2)	12 (36.4)
MD (n = 24 )	3 (12.5)	9 (37.5)	4 (16.7)	8 (33.3)
Kruskal-Wallis H, p-value	0.09	0.07	0.08	0.07
**Sugar consumption**				
Yes (n = 329)	17 (5.2)	147 (44.7)	66 (20.0)	99 (30.1)
No (n = 71)	6 (8.4)	44 (61.9)	11 (15.6)	10 (14.1)
Chi-squared test, p-value	0.07	0.06	0.06	0.04

CP: cerebral palsy; A: autism; DS: Down’s syndrome; ID: intellectual disability; DB: deafness or blindness or both; MD: children with multiple disabilities, syndromes.

Caries was present in 289 (72.3%) with a mean dmft of 3.9 ± 2.7, and mean DMFT of 4.8 ± 2.3 (Table 2).

**Table 2:** Mean (standard deviation) caries scores according to age

Variables	n	dt	dmft	ds	dmfs	DT	DMFT	DS	DMFS
**Age in years**									
6–11	160	3.1 (2.1)	3.9 (2.7)	5.1 (3.1)	5.7 (4.1)	0.7 (0.3)	0.9 (0.4)	1.1 (0.8)	1.7 (1.1)
12–16	240	0.02 (0.01)	0.02 (0.01)	0.05 (0.03)	0.07 (0.02)	3.2 (1.9)	3.9 (2.1)	4.2 (2.7)	5.6 (2.8)
p-value*		0.02	0.03	0.001	0.001	0.03	0.04	0.001	0.001

Values in parenthesis shows standard deviation. *t-test.

Regression analysis for obesity showed an odds ratio (OR) of 1.69 (CI: 0.18–2.62, p < 0.05), and for children with sugar consumption 2.01 (CI: 0.18–3.09, p < 0.05) for having caries. The OR for cerebral palsy was 2.21 (CI: 1.27–4.12, p < 0.001) and for intellectual disability 2.27 (CI: 1.29–5.12, p < 0.001) for having caries (Table 3).

**Table 3:** Association between dependent variable caries with the independent variables examined

Variable	Caries n	Caries %	Non-adjusted ORs (CI)	Adjusted ORs (CI)+
**Age in years**				
6-11 (n = 160)	129	80.6	0.93 (0.09–1.92)	0.95 (0.11–1.98)
12-16 (n = 240) #	160	66.7		
**Gender**				
Boys (n = 180) #	118	65.6		
Girls (n = 220)	171	77.7	0.87 (0.02–1.62)	0.89 (0.03–1.69)
**Type of disability**				
CP (n = 43)	33	76.7	2.19 (1.21–4.04)**	2.21 (1.27–4.12)**
A (n = 107)	84	78.5	2.15 (1.14–4.17)**	2.17 (1.18–4.23)**
DS (n = 70)	33	47.1	0.64 (0.03–1.08)	0.71 (0.07–1.11)
ID (n = 123)	98	79.7	2.24 (1.21–5.08)**	2.27 (1.29–5.12)**
DB (n = 33)	22	66.7	1.12 (0.21–1.89)	1.19 (0.32–1.93)
MD (n = 24)	19	79.2	2.25 (1.27–5.14)**	2.29 (1.31– 5.21)**
**BMI**				
Underweight and normal weight (n = 214) #	144	67.3		
Overweight and obese (n = 186)	145	77.9	1.62 (0.14–2.53)*	1.69 (0.18–2.62)*
**Sugar consumption**				
Yes (n = 329)	249	75.7	1.98 (0.13–3.02)*	2.01 (0.18–3.09)*
No (n = 71) #	40	56.3		
**Oral hygiene habits**				
Toothbrushing frequency				
≤ 1 times/day (n = 183)	142	77.6	1.96 (0.12–3.09)*	2.02 (0.18–3.12)*
≥ 2 times/day (n = 217) #	147	67.7		
**Fluoridated dentifrice**				
Yes (n = 234) #	152	64.9		
No and don’t know (n = 166)	137	82.5	2.63 (0.74–4.93)**	2.67 (0.76–5.03)**

# Reference; *p < 0.05, **p < 0.001; CI: Confidence interval; +adjusted for age, gender, type of disability, BMI, sugar consumption, and oral hygiene practices. CP: cerebral palsy; A: autism; DS: Down’s syndrome; ID: intellectual disability; DB: deafness or blindness or both; MD: children with multiples disability, syndromes. For disability types, children with other disabilities are taken as reference value for each disability type.

## Discussion

The present study was the first of its kind, which examined the relationship between caries and obesity among children with special health-care needs controlling covariates like gender, age, type of disability, diet, and oral hygiene practices. The results showed 72.3% caries prevalence with high mean caries scores (dmft 3.9; DMFT 4.8). The caries prevalence reported in the present study is closer to Saudi National Survey data.^13^ No significant difference was observed between gender and caries prevalence.

The present study found a high prevalence of caries among children with cerebral palsy (76.7%), intellectual disability (79.7%), autism (78.5%), and children with multiple disabilities (79.2). Children with cerebral palsy had an OR of 2.21, autism an OR of 2.17, intellectual disability an OR of 2.27, and multiple disabilities an OR of 2.29 greater risk of having caries. The high prevalence and greater odds of developing caries in these special-needs children may be attributed to lack of motor coordination, psychomotor function impairment, and lack of manual dexterity, which directly influence the practice of oral hygiene measures.^7,8,16,20,24^

The current study’s results showed a positive association between caries and obesity with an OR of 1.69 greater risk of caries compared to children with normal BMI. This finding is in agreement with previous studies and systematic reviews which showed a strong association between the two.^5,7,11,12,15,19^ The reason may be due to the effect of the common risk factor – sugar consumption – which increased the likelihood of both conditions. The current results showed obesity to be statistically significantly higher among special-needs children who regularly consumed sugar; these children were also at a 2.01-times greater risk of having caries.

In agreement with previous studies,^10,25^ the present findings showed that children who brushed their teeth ≤ 1 per day had a 2.02-times greater risk of having caries. Children who used non-fluoridated toothpaste presented with 82% caries prevalence and a 2.67-times greater risk of having caries. These results demonstrated the need to establish measures promoting habits for adequate oral hygiene with the proper use of fluoridated dentifrices in special-needs children.^23^

The cross-sectional design of the present study limits the establishment of a causation relationship; other unexplored facors might also have influenced the observed association. Chances of recall bias in diet history may also have influenced the study results.

## Conclusion

The present study showed a positive association between caries and obesity among children with special health-care needs. This observation demands an integrated approach to caries management by addressing the common risk factors for caris and obesity, thus emphasising both oral and general health of special-needs children.
